# Flexible High-Frequency Underwater Transducer Based on Piezoelectric Composites

**DOI:** 10.3390/mi17050577

**Published:** 2026-05-07

**Authors:** He Zhou, Chao Zhong, Lei Qin

**Affiliations:** Beijing Key Laboratory for Sensors, Beijing Information Science & Technology University, Beijing 100192, China; 15524802673@163.com

**Keywords:** flexible transducers, high-frequency underwater transducers, piezoelectric composites, same-side electrodes

## Abstract

In this study, a flexible, lightweight high-frequency underwater transducer (FT) was designed and fabricated. To ensure the flexibility and reliability of the transducer, a flexible piezoelectric composite material with a same-side electrode configuration and a perforated flexible printed circuit (FPC) were designed. First, finite element simulation analysis was performed on the flexible piezoelectric composites to optimize the structural parameters. Next, using a cutting and infusion method combined with reflow soldering, the piezoelectric composites and FPC were integrated to form a flexible sensing element. Finally, a flexible packaging process for the underwater transducer was investigated, resulting in a flexible underwater transducer with a thickness of only 5.3 mm. The results of underwater electroacoustic comparison tests show that the transmission and reception performance of the FT differs by less than 0.5 dB from that of conventional rigid transducers. This demonstrates that the flexible underwater transducer developed in this study not only possesses the advantages of flexibility and can be conformally mounted on curved surfaces but also achieves acoustic performance comparable to that of traditional rigid transducers, thereby providing new insights for the lightweight development of underwater transducers.

## 1. Introduction

Underwater transducers are the core components for transmitting or receiving sound waves in sonar systems [1,2,3], and they enable underwater detection, communication, and imaging [4]. Based on operating frequency, underwater transducers can be broadly classified into three categories: high-frequency, mid-frequency, and low-frequency. High-frequency underwater transducers typically operate at frequencies of several hundred kHz. They are commonly used in small platforms such as unmanned underwater vehicles (UUVs) and imaging sonars [5,6], which generally have limited internal space and payload capacity. High-frequency underwater transducers typically consist of a piezoelectric element, a rigid foam backing, positive and negative leads, and a housing. Their sensing elements are often made of piezoelectric ceramics, piezoelectric single crystals, or piezoelectric composites [7]. Piezoelectric composites are formed by combining a piezoelectric phase (such as piezoelectric ceramics or single crystals) with a polymer phase in a specific interconnection configuration, with the 2-2 and 1-3 types being the most commonly used [8]. When using piezoelectric composites as the sensing element, transducers typically achieve a higher transmission voltage response (*TVR*) and receive voltage sensitivity (*M*) [9,10,11,12,13,14]. However, current high-frequency underwater transducers are primarily rigid bulk structures; once encapsulated and molded, their shape is difficult to alter [15]. When integrated into small underwater platforms, conventional rigid transducers occupy valuable internal space, are difficult to conformally mount on curved surfaces, and limit system portability. Therefore, the development of an underwater transducer that is flexible, lightweight, and capable of being conformally mounted on the carrier surface holds promise for resolving these issues.

In recent years, advances in flexible electronic technology have provided new solutions for the development of flexible transducers [16,17,18]. This technology integrates functional materials or components onto flexible substrates, thereby creating transducers with flexible or stretchable properties. In particular, significant research progress has been made in the development of flexible ultrasonic transducers for medical imaging and health monitoring [19,20,21]. For example, the University of Southern California developed a wearable ultrasound transducer [22], which consists of a laminated structure comprising a backing layer, a flexible printed circuit board (FPC), a piezoelectric composite array, a matching layer, and a bioadhesive coupling agent and can be used for medical ultrasound imaging. The University of California, San Diego has also developed a flexible ultrasonic transducer [23]. This transducer employs serpentine “island-bridge” electrodes encapsulated in a thin layer of silicone resin, endowing the transducer with stretchable properties. However, current flexible transducers face challenges in underwater applications for the following reasons: First, current flexible transducers are designed for applications such as medical ultrasound and industrial non-destructive testing, where operating frequencies are typically in the MHz range, making it difficult to meet the requirements of underwater acoustic detection, which operates at frequencies of several hundred kHz. Second, these flexible transducers mostly employ serpentine “island-bridge” electrodes, thin-film stretchable electrodes, or soft-substrate packaging structures. Their long-term reliability and pressure tolerance in underwater environments remain challenging. To address the above issues, this study proposes a flexible piezoelectric composite material with same-side electrodes and, based on this, develops a high-frequency underwater transducer capable of operating underwater.

## 2. Transducer Structure

**Figure 1:**
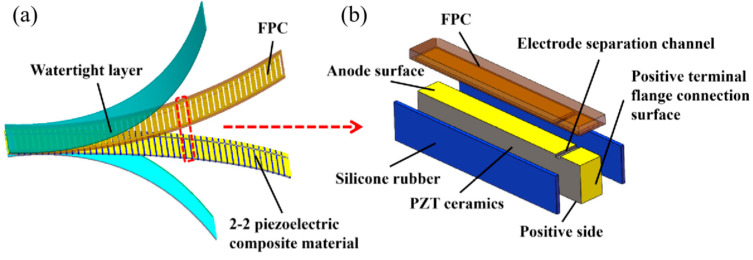
Structure of a flexible high-frequency underwater transducer: (**a**) overall structure; (**b**) single-cycle structure.

The structure of the flexible high-frequency underwater transducer (FT) is shown in Figure 1a and consists of three components: a Type 2-2 piezoelectric composite, an FPC, and a watertight layer. The Type 2-2 flexible piezoelectric composite is composed of piezoelectric columns and silicone rubber arranged in a periodic alternating pattern. The stretchability of the silicone rubber provides the composite with sufficient flexibility. As shown in Figure 1b, to ensure the flexibility of the transducer and the reliability of the electrical connections, this study employs same-side electrodes design in piezoelectric composites, combined with an FPC film electrode featuring a perforated structure where the positive and negative electrodes are connected in a common configuration. This ensures reliable internal connection between the positive and negative electrodes within the sensing element while allowing both electrodes to be led out from the same side, thereby guaranteeing the flexibility of the device after encapsulation and enabling conformal mounting on curved surfaces. This design addresses the issue of insufficient reliability in serpentine “island-bridge” structures when used underwater while also simplifying the fabrication process. Although this structure appears to be an array, all ceramic column positive electrodes are connected via the FPC, and the negative electrodes are also interconnected; therefore, it can still be considered a flexible piezoelectric composite. The transducer studied in this study must both transmit and receive sound waves. Among commonly used PZT-series piezoelectric ceramics, PZT-5A possesses a high electromechanical coupling coefficient *k*_33_ (approximately 0.71) and a high piezoelectric strain constant *d*_33_ (approximately 400 pC/N). These properties facilitate the achievement of superior combined transmission and reception performance, and thus it was selected as the material for the piezoelectric columns [24]. Silicone rubber type 704 was selected for its good tensile properties and high tear strength (approximately 21–31 N/mm), which ensure the structural flexibility and strength of the transducer [25]. The 2-2-type flexible piezoelectric composite serves as the sensing element responsible for acoustic-to-electric conversion in this transducer and determines its performance. Therefore, this study focuses on its simulation analysis to optimize structural parameters and enhance the transducer’s performance.

## 3. Finite Element Analysis

**Figure 2:**
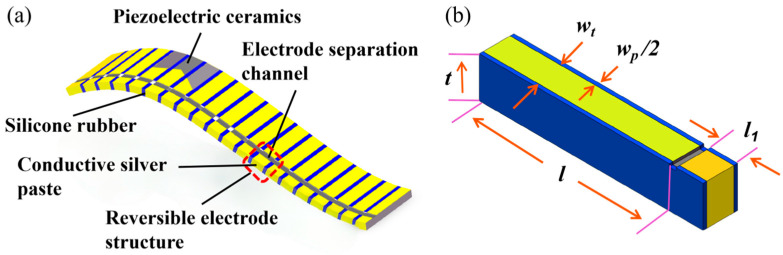
Type 2-2 Flexible piezoelectric composite with the same-side electrode structure and COMSOL modeling: (**a**) Type 2-2 flexible piezoelectric composite; (**b**) modeling a single cycle in COMSOL.

As shown in Figure 2a, the flexible Type 2-2 piezoelectric composite exhibits periodic repetition. To balance computational efficiency and accuracy, a single periodic element (Figure 2b) was used to establish the finite element model in the simulation. This periodic cell consists of a piezoelectric ceramic column flanked by silicone rubber on both sides, where t is the thickness of the piezoelectric composites, *w_t_* is the width of the piezoelectric column, *w_p_* is the width of the silicone rubber, *l* is the effective radiating surface length of the piezoelectric column, *l*_1_ is the length of the same-side electrode, and the dimensions of the groove separating the positive and negative electrodes are 0.5 mm (width) × 0.5 mm (depth).

Finite element analysis was performed using the Frequency Domain module in COMSOL 6.3. In the simulation, the piezoelectric ceramic PZT-5A uses parameters from COMSOL’s built-in material library, while the material parameters for silicone rubber are shown in Table 1 [25]. An alternating voltage excitation is applied between the positive and negative electrodes of the piezoelectric ceramic. Using frequency-domain sweep analysis, the steady-state electromechanical coupling response of the piezoelectric composites under alternating excitation is solved, thereby obtaining frequency response parameters such as the admittance or impedance of the piezoelectric composites.

Admittance curves are primarily used to characterize the resonant response of piezoelectric composites and can be employed to determine whether the principal vibration mode is pure [26]. From the admittance curve, the resonance frequency *f_r_* corresponding to the maximum point and the anti-resonance frequency *f_a_* corresponding to the minimum point can be extracted. Equation (1) can be used to calculate the effective electromechanical coupling coefficient (*k_eff_*) of the composite material. *k_eff_* is a key parameter for evaluating the electromechanical conversion capability of the composite material; generally, the larger the value of *k_eff_*, the higher the electromechanical conversion efficiency of the transducer [27,28]. Therefore, the effects of the composite thickness *t*, the effective radiating surface length of the piezoelectric column *l*, the ceramic volume fraction *v*, and the same-side electrode length *l*_1_ on the resonance characteristics were investigated through finite element simulations. Structural parameters for flexible piezoelectric composites were designed based on the obtained patterns.


(1) $$k_{eff}=\sqrt{\frac{f_{a}^{2}-f_{r}^{2}}{f_{a}^{2}}}$$

The volume fraction *v* of the piezoelectric ceramic refers to the proportion of the piezoelectric ceramic by volume in piezoelectric composites and can be expressed as

(2) $$v=\frac{w_{t}}{w_{t}+w_{p}}$$

### 3.1. Effect of Composite Thickness t on Resonance Performance

**Figure 3:**
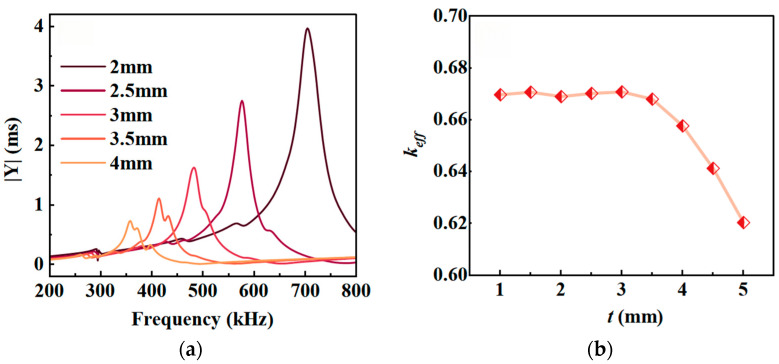
Curves showing the variation in resonant performance with *t*: (**a**) admittance; (**b**) electromechanical coupling coefficient *k_eff_*.

The transducer developed in this study utilizes the thickness vibration mode of flexible piezoelectric composites, and the resonant frequency of the thickness vibration primarily depends on the thickness *t* of the composites [29]. Here, with *l* = 14 mm, *v* = 65%, and *l*_1_ = 2 mm fixed, simulation calculations were performed to obtain the admittance curves for different *t* values, and the corresponding electromechanical coupling coefficient *k_eff_* was calculated. The results are shown in Figure 3.

Figure 3a shows that when the length and width of the piezoelectric column are fixed, the smaller *t*, the higher the resonant frequency *f_r_* of the flexible piezoelectric composites and the larger the modulus of admittance. This pattern is consistent with the characteristics of thickness vibrations in piezoelectric materials. As shown in Figure 3b, *k_eff_* remains around 0.67 within the thickness range of 1 mm to 3.5 mm; however, when the *t* value exceeds 3.5 mm, the electromechanical coupling coefficient exhibits a noticeable downward trend. This is primarily due to interference from other vibration modes near the thickness resonance. As shown by the admittance curves for *t* = 3.5 and *t* = 4 mm in Figure 3a, two distinct resonance peaks appear in the admittance curve.

### 3.2. Effect of the Volume Fraction v of Piezoelectric Ceramics on Resonance Performance

**Figure 4:**
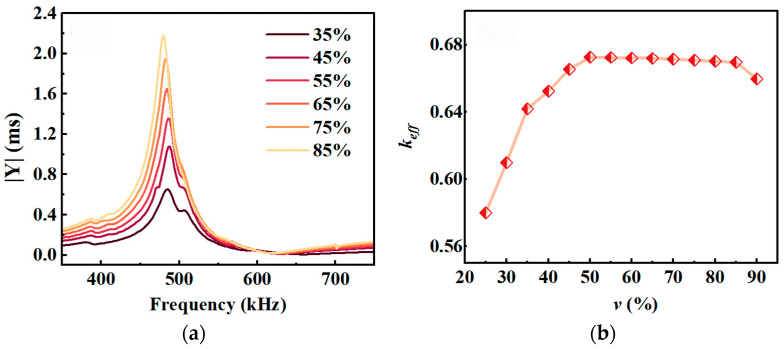
Curves showing the variation in resonant performance with *v*: (**a**) admittance; (**b**) electromechanical coupling coefficient *k_eff_*.

The sum of the ceramic column width *w_t_* and the silicone rubber width *w_p_* was set to 1.55 mm. With *t* = 3 mm, *l* = 14 mm, and *l*_1_ = 2 mm, the *v* value was adjusted by varying *w_t_* and *w_p_*. The simulation results for different *v* values are shown in Figure 4.

Figure 4a shows that the modulus of the admittance of piezoelectric composites gradually increases as the volume fraction of the piezoelectric ceramic increases. Figure 4b shows that as *v* increases, *k_eff_* rises from 0.58 to approximately 0.67 in the range of 25% to 45%; in the range of 45% to 85%, *k_eff_* remains essentially constant; and when *v* exceeds 85%, *k_eff_* gradually decreases. Therefore, to achieve high electromechanical conversion efficiency, *v* should be within the range of 45% to 85%.

### 3.3. Effect of the Effective Radiating Surface Length l of the Piezoelectric Column on Resonance Performance

**Figure 5:**
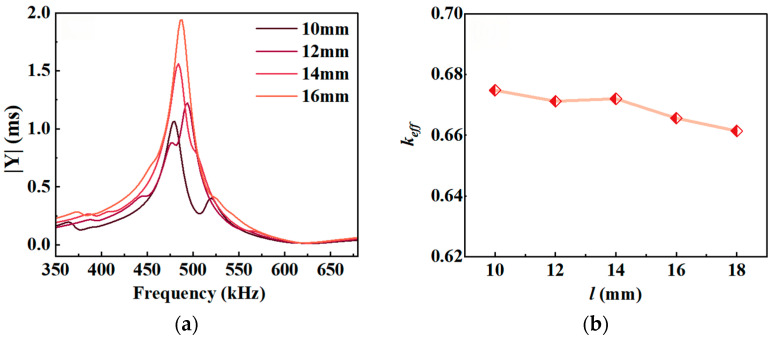
Resonance characteristics as a function of *l*: (**a**) admittance; (**b**) electromechanical coupling coefficient *k_eff_*.

With *t* = 3 mm, *v* = 65%, and *l*_1_ = 2 mm held constant, simulations were performed for *l* values of 10, 12, 14, mm, and 18 and 18 and 18,. The results are shown in Figure 5.

Figure 5a shows that as *l* increases, the admittance exhibits only a single resonance peak across the frequency sweep range. When *l* is too small, such as *l* = 12 mm and 10 mm, two closely spaced resonance peaks appear in the admittance curve, indicating interference from other vibrations. Figure 5b shows that the electromechanical coupling coefficient *k_eff_* exhibits a slight downward trend as *l* increases. Therefore, to ensure that the composite material exhibits pure thickness vibration, the length *l* of the piezoelectric column should be greater than 12 mm, but it should not be excessively long.

### 3.4. Effect of Same-Side Electrode Length l_1_ on Resonance Performance

**Figure 6:**
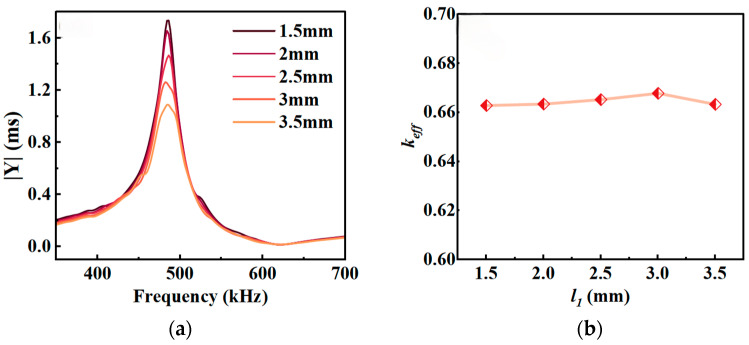
Resonance characteristics as a function of *l*_1_: (**a**) admittance; (**b**) electromechanical coupling coefficient *k_eff_*.

To achieve simultaneous lead-out of the positive and negative electrodes on the same side, same-side electrodes were incorporated on one side of the composite material in this study. To analyze the effect of *l*_1_ on the resonance performance of the composite material, simulations were performed for composites with different values of *l*_1_ while keeping *t* = 3 mm, *l* = 14 mm, and *v* = 65% constant. The results are shown in Figure 6.

Figure 6a shows that variations in *l*_1_ have virtually no effect on *f_r_*, and the admittance curve exhibits a single resonance peak throughout the swept frequency range, indicating that changes in *l*_1_ do not induce significant lateral coupling vibrations. Figure 6b shows that changes in *l*_1_ have only a slight effect on the electromechanical coupling coefficient *k_eff_* of the composite material. Although a smaller *l*_1_ results in a higher admittance modulus, this reduces the reliability of the connection between the FPC and the ceramic electrode. Therefore, considering both resonance performance and soldering reliability, an *l*_1_ of approximately 2 mm is appropriate for the same-side electrode.

Based on the aforementioned patterns of the resonance performance of the flexible piezoelectric composites as a function of structural parameters and taking into account the flexibility of the transducer, the composite material thickness was designed to be *t* = 3 mm, the effective radiating surface length of the piezoelectric column to be *l* = 14 mm, the length of the same-side electrode to be *l*_1_ = 2 mm, and the width of the groove separating the positive and negative electrodes to be 0.5 mm. The volume fraction of the piezoelectric ceramic is *v* = 0.65, corresponding to a piezoelectric column width of *w_t_* = 1.0 mm and a silicone rubber width of *w_p_* = 0.55 mm.

## 4. Fabrication and Testing of Flexible High-Frequency Underwater Transducers

### 4.1. Preparation of Flexible Sensing Elements

**Figure 7:**
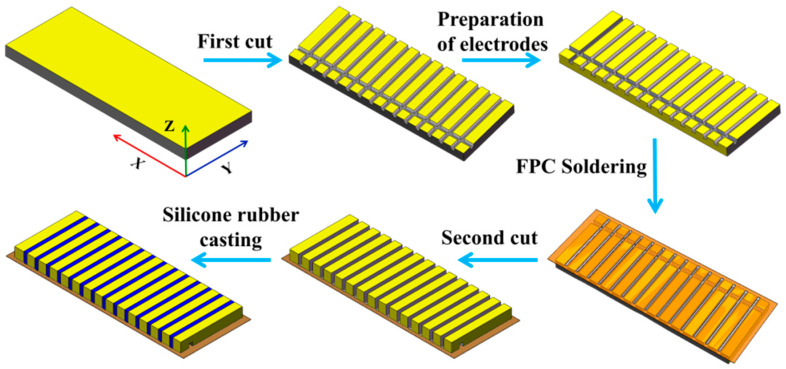
Fabrication process for flexible sensing elements.

A “soldering–dicing–encapsulation” process was used to integrate the piezoelectric ceramic phase, FPC electrodes, and flexible polymer phase into a flexible sensing element. This process follows a sequence of “welding the FPC first, then casting silicone rubber” which prevents pre-formed piezoelectric composites from deforming due to heat during the FPC welding process. This helps maintain electrical connection reliability and reduces the difficulty of subsequent encapsulation. The specific fabrication process is shown in Figure 7.

**Figure 8:**
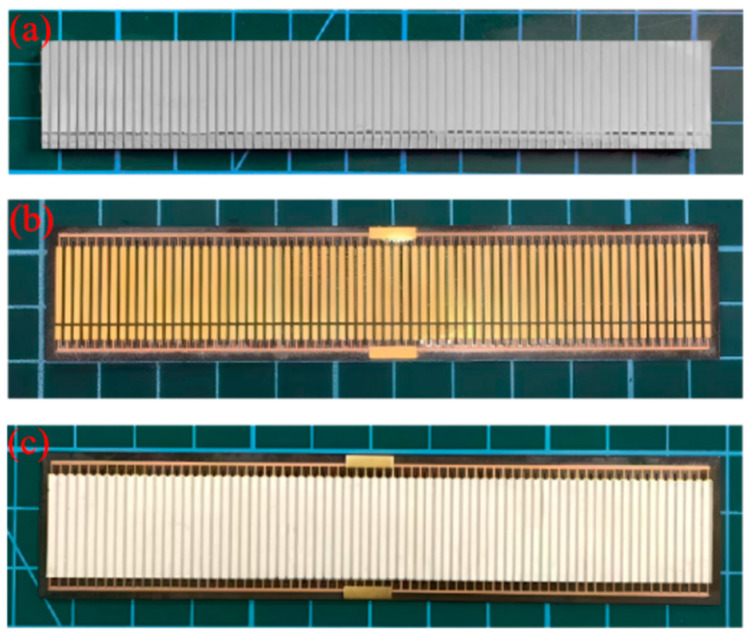
Flexible sensing elements with same-side electrodes: (**a**) piezoelectric column array; (**b**) FPC; (**c**) flexible piezoelectric sensing element.

(1) Initial dicing: We cut the negative electrode surface of a rectangular piezoelectric ceramic wafer (111.5 mm × 16.5 mm × 3 mm). First, we cut along the Y direction to form an array of piezoelectric pillars with a ceramic substrate (step size 1.55 mm, cutting depth 1 mm, kerf 0.55 mm). Then, we cut an anode-cathode separation groove along the X direction, with dimensions of 0.5 mm (width) × 0.5 mm (depth). A photograph of the cut piezoelectric column array is shown in Figure 8a.

(2) Preparation of same-side electrodes: Using conductive silver paste, the positive surface of the piezoelectric ceramic is connected through the side to the negative side to form the same-side electrode structure.

(3) Soldering the FPC: The FPC (115 mm × 22 mm × 0.25 mm) was soldered to the piezoelectric column array using a reflow soldering process. The FPC is shown in Figure 8b.

(4) Back-side dicing: We cut the ceramic anode surface (i.e., the ceramic substrate) only in the Y direction. The step size and kerf width are the same as in Step (1), and we ensure that the kerf extends all the way through from top to bottom.

(5) Silicone rubber casting: We pour the silicone rubber onto the anode surface, evacuate the chamber to remove air bubbles, and cure it at room temperature for 24 h. The resulting flexible piezoelectric sensing elements measure 115 mm × 22 mm × 3.3 mm, as shown in Figure 8c.

### 4.2. Fabrication of Flexible Underwater Transducers

**Figure 9:**
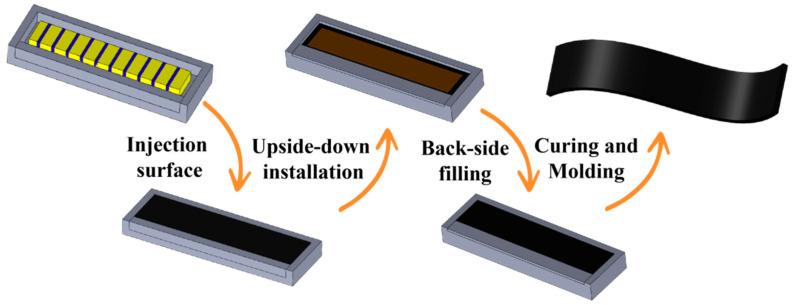
Encapsulation process for flexible underwater transducers.

**Figure 10:**
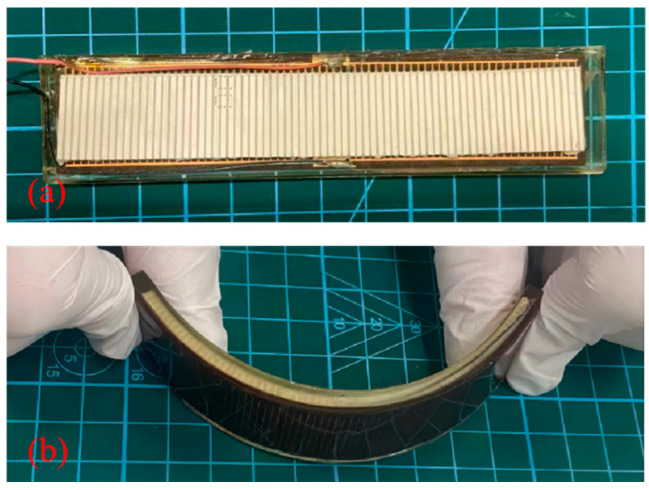
Flexible transducer (FT): (**a**) planar configuration; (**b**) curved configuration.

The encapsulation material selected for the transducers is common polyurethane (AJ-2S). This polyurethane material has a sound transmission coefficient > 0.9 and a Shore A hardness of approximately 70, which not only reduces sound transmission loss in the encapsulation layer but also ensures that the transducer possesses high flexibility [30]. The encapsulation of the flexible underwater transducer is performed by first encapsulating the radiating surface and then the back surface, as shown in Figure 9. The thickness of the watertight layer on both the radiating surface and the back surface is 1 mm. The encapsulated flexible underwater transducer is shown in Figure 10. The overall dimensions of the transducer are 120 mm (length) × 25 mm (width) × 5.3 mm (thick).

### 4.3. Comparative Testing of Flexible Underwater Transducers

To verify the acoustic transmission and reception performance of the developed flexible transducer, this study conducted comparative underwater acoustic performance tests between the flexible transducer (FT) and control group devices, including a conventional rigid planar transducer (SPT) and a rigid curved transducer (SCT). The tests included measurements of the transmission voltage response (*TVR*) and receiving voltage sensitivity (*M*), both of which were performed using the pulse method in a water tank [31,32].

**Figure 11:**
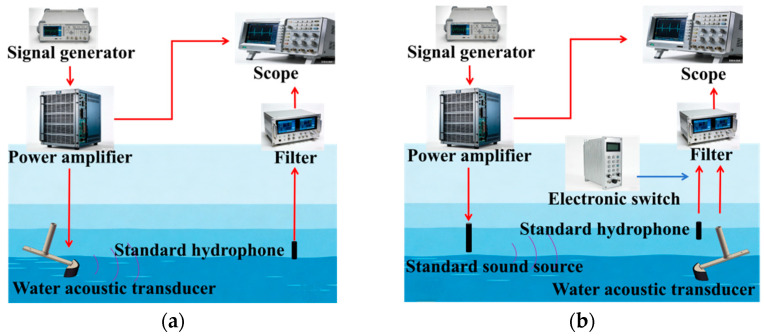
Pulse-based underwater acoustic detection equipment: (**a**) transmission voltage response (*TVR*) test; (**b**) receiving voltage sensitivity (*M*) test.

The transmission voltage response (*TVR*) is a key parameter characterizing the transmission performance of an underwater transducer, reflecting the transducer’s electro-acoustic conversion capability in transmission mode. A higher *TVR* indicates that the transducer can produce a higher sound pressure output under the same excitation voltage. When testing using the pulse method, with the experimental setup shown in Figure 11a, the *TVR* of the transducer under test can be expressed as

(3) $$TVR=20\mathrm{lg}(\frac{dU_{p}}{U_{x}})-M_{oL}$$

where *d* is the acoustic center-to-center distance between the transducer under test and the reference hydrophone, *U_x_* is the input voltage of the transducer under test, *U_p_* is the open-circuit voltage received by the reference hydrophone, and *M_oL_* is the known sensitivity level of the reference hydrophone.

Receiving voltage sensitivity (*M*) is a core parameter characterizing the receiving performance of an underwater transducer; it is used to measure the transducer’s ability to convert incident sound pressure into an electrical signal in receive mode. A higher value of *M* indicates that the transducer can output a stronger electrical signal under the same sound pressure. The equipment layout when using the pulse method for testing is shown in Figure 11b, and the receiving voltage sensitivity of the transducer under test can be expressed as:

(4) $$M=20\mathrm{lg}(\frac{U_{x}d_{x}}{U_{o}d_{o}})-M_{oL}$$

where *d_o_* and *d_x_* are the acoustic center distances from the standard sound source to the standard hydrophone and the transducer under test, respectively, and *U_x_* and *U_o_* are the open-circuit output voltages of the standard hydrophone and the transducer under test, respectively.

**Figure 12:**
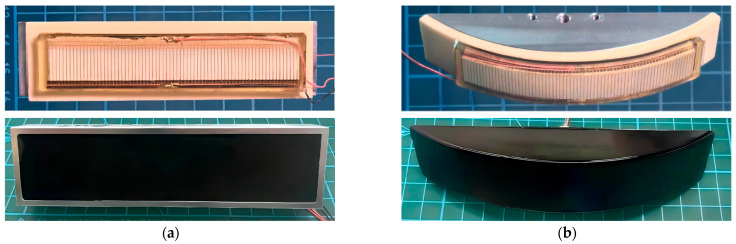
Comparative testing of flexible and rigid transducers under two different conditions: (**a**) a flexible transducer (FT) conformally mounted on a planner surface compared to a rigid planar transducer (SPT); (**b**) a flexible transducer (FT) conformally mounted on a curved surface compared to a rigid curved transducer (SCT).

To validate the comparative measurements, the FT was tested under two mounting conditions: planar conformal mounting and curved conformal mounting. Photographs of the rigid planar transducer (SPT) and the rigid curved transducer (SCT) are shown in Figure 12, with the SCT having a diameter of 200 mm. The materials and structural parameters of their sensing elements were identical to those of the FT. Photographs of the flexible transducers mounted on their respective housings are shown in Figure 12.

**Figure 13:**
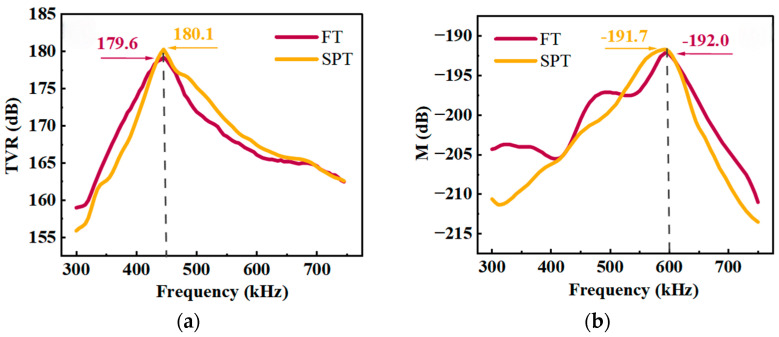
Test results for planar conformally mounting: (**a**) TVR; (**b**) M.

Figure 13 shows the transmission voltage response and receiving voltage sensitivity of the FT under planar conformally mounted conditions, compared with the test results of the SPT. Figure 13a shows that the resonance frequencies of both the FT and the SPT during transmission are approximately 450 kHz, with the FT’s peak transmission voltage response at 179.6 dB and the SPT’s at 180.1 dB—a difference of only 0.5 dB. Figure 13b shows that the peak receiving voltage sensitivity of both the FT and the SPT corresponds to a frequency of approximately 600 kHz, with the FT’s peak receiving voltage sensitivity at −192.0 dB and the SPT’s at −191.7 dB, a difference of only 0.3 dB.

**Figure 14:**
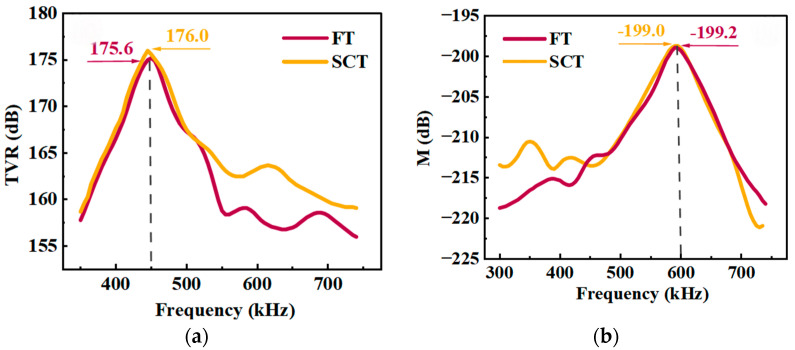
Test results for curved conformally mounting: (**a**) TVR; (**b**) M.

Figure 14 shows the results of a comparative test between the FT under curved conformally mounted conditions and the SCT. Figure 14a shows the transmission voltage response curves; both the FT and the SCT peak at around 450 kHz, with the FT reaching a peak of 175.6 dB and the SCT reaching 176.0 dB—a difference of only 0.4 dB. Figure 14b shows the receiving voltage sensitivity curves; the peaks of both are also near 600 kHz, with the FT’s receiving voltage sensitivity peaking at −199.2 dB and the SCT’s at −199.0 dB, a difference of only 0.2 dB.

## 5. Conclusions and Discussion

This study describes the design and fabrication of a flexible thin-layer high-frequency underwater transducer (FT) with a total thickness of only 5.3 mm. Results from underwater electroacoustic comparison tests show that, when conformally mounted on a flat surface, the FT’s transmission voltage response and receiving voltage sensitivity differ from those of a conventional rigid planar transducer (SPT) by only 0.5 dB and 0.3 dB, respectively. When conformally mounted on a curved surface, the FT’s transmission voltage response and receiving voltage sensitivity differed from those of a conventional rigid curved-surface transducer (SCT) by only 0.4 dB and 0.2 dB, respectively. This indicates that the flexible high-frequency underwater transducer proposed in this study not only offers the advantages of conformal mounting but also maintains acoustic performance very close to that of conventional rigid transducers.

Although the transducer (FT) developed in this study is flexible, its encapsulation material (polyurethane) is identical to that used in traditional rigid underwater transducers. Theoretically, the transducer’s transmission voltage response primarily depends on the thickness of the piezoelectric composites, the equivalent piezoelectric strain constant *d*_33_, and the transducer’s shape, dimensions, and effective radiating area. The reception voltage sensitivity primarily depends on the thickness of the piezoelectric composites and the piezoelectric voltage constant *g*_33_. Since the FT, SPT, and SCT all utilize piezoelectric composites with identical parameters and the FT exhibits similar geometries to the control group (SPT and SCT) in both planar and curved conformal states, the flexible underwater transducer developed in this study shows no significant difference in acoustic performance compared to traditional rigid underwater transducers.

## Figures and Tables

**Table 1:** Material properties of silicone rubber.

Parameter	Value	Unit
*ρ*	1100	kg/m^3^
*c*	1000	m/s
*E*	1 × 10^6^	Pa
*σ*	0.48	1

## Data Availability

The data presented in this study are available within the article.
